# Overexpression of *EcbHLH57* Transcription Factor from *Eleusine coracana* L. in Tobacco Confers Tolerance to Salt, Oxidative and Drought Stress

**DOI:** 10.1371/journal.pone.0137098

**Published:** 2015-09-14

**Authors:** K. C. Babitha, Ramu S. Vemanna, Karaba N. Nataraja, M. Udayakumar

**Affiliations:** Department of Crop Physiology, University of Agricultural Sciences, Bangalore, Karnataka, India; Indiana University, UNITED STATES

## Abstract

Basic helix-loop-helix (bHLH) transcription factors constitute one of the largest families in plants and are known to be involved in various developmental processes and stress tolerance. We report the characterization of a stress responsive bHLH transcription factor from stress adapted species finger millet which is homologous to OsbHLH57 and designated as EcbHLH57. The full length sequence of EcbHLH57 consisted of 256 amino acids with a conserved bHLH domain followed by leucine repeats. In finger millet, *EcbHLH57* transcripts were induced by ABA, NaCl, PEG, methyl viologen (MV) treatments and drought stress. Overexpression of *EcbHLH57* in tobacco significantly increased the tolerance to salinity and drought stress with improved root growth. Transgenic plants showed higher photosynthetic rate and stomatal conductance under drought stress that resulted in higher biomass. Under long-term salinity stress, the transgenic plants accumulated higher seed weight/pod and pod number. The transgenic plants were also tolerant to oxidative stress and showed less accumulation of H_2_0_2_ and MDA levels. The overexpression of *EcbHLH57* enhanced the expression of stress responsive genes such as *LEA14*, *rd29A*, *rd29B*, *SOD*, *APX*, *ADH1*, *HSP70* and also *PP2C* and hence improved tolerance to diverse stresses.

## Introduction

The environmental stress signals such as drought, salinity, high temperature perceived by the plants results in diverse stress responses. These responses are regulated by many complex mechanisms which are controlled by transcriptional regulation. Transcription factors (TFs) play important regulatory roles in stress responses by regulating their target gene expression by binding to the conserved cis-acting elements [1, 2, 3]. Several stress responsive TFs belonging to APETELA2 (AP2), bHLH, bZIP, NAC, ZF, MYB and WRKY families have been well elucidated for their regulatory roles under various stress conditions. Transgenic plants overexpressing these groups of genes have shown improved tolerance to different stresses [4, 5, 6, 7].

The bHLH proteins are one of the largest families of transcription factors both in animals and plants and are well characterized in mammalian system [8]. The bHLH domain contains approximately 60 amino acids, with two functionally distinctive regions, the basic region and the HLH region. The basic region consists of 15 amino acids and functions as a DNA binding domain and HLH region contains two amphipathic α- helices linked by a loop. This allows the formation of homodimers or heterodimers [9, 10]. bHLH proteins bind to sequences containing a consensus core elements of the E-box (5’-CANNTG-3’), and the G box (5’-CACGTG-3’) cis elements. In *Arabidopsis* and rice, 162 and 167 *bHLH* encoding genes have been identified respectively [11, 12]. Many of these genes have been characterized for their role in developmental processes such as flavonoid and anthocyanin biosynthesis [10, 13], trichome and tapetum development [14, 15], shoot branching [16], controlling grain length and width [17, 18]. Some of the genes are also involved in signaling processes [19, 20, 21]. A number of *bHLH* genes have been shown to be involved in stress tolerance. The microarray analysis of NaCl treated *Arabidopsis* roots showed a differential regulation of 29 *bHLH* genes [22]. In soybean, out of 45 *bHLH* genes, 14 are responsive to abiotic/biotic stresses. Improved tolerance to diverse abiotic stresses has been reported in transgenic plants overexpressing *AtbHLH92* [22], *AtICE1* [23], *OsbHLH1* [24], *MdCIbHLH1* [25]. Both, *AtICE1* in *Arabidopsis* and *MdCIbHLH1* in apple enhanced the expression of C-repeat binding factor (CBF) regulons.

Across the species there is a significant variation in the threshold levels of tolerance. One of the reasons attributed was the stress responsive functional or regulatory gene transcripts/proteins from tolerant species are either stable or functionally more efficient under stress [26, 3]. Comparative transcriptome studies in susceptible and tolerant rice varieties showed upregulation of 1900 genes with 77 TFs in drought tolerant N22 genotype as compared to drought susceptible IR64 variety which showed significant downregulation of regulatory genes. Hence tolerant variety may have better mechanisms to reprogram the expression of genes under stress conditions [27]. Similarly superoxide dismutase from drought tolerant *Arabidopsis thaliana* ecotype Cvi showed better stability under high photo-oxidative stress than from drought susceptible ecotype *ler* [28]. Hence stress tolerant genotype/species may have better alleles which may contribute to survival under adverse climatic conditions [27]. Hence recent research focus has been on validating genes from stress adapted species such as such as *Thellungiella halophila*, a salt-tolerant relative of *Arabidopsis*, *Atriplex hortensis* and *Tamarix hispida* due to their extreme stress adaptive nature [29, 30, 31, 32]. Overexpression of *OrbHLH001*, a close homolog of ICE1 from wild rice *Oryza rufipogan* in *Arabidopsis* resulted in higher salinity tolerance [33]. Even *OrbHLH2* also showed increased tolerance to salt and osmotic stress by upregulating several dehydrin group of genes [34]. We also showed that overexpression of *EcNAC1* transcription factor from finger millet in tobacco improved the tolerance to abiotic stresses [35]. In the present study our focus is to characterize a bHLH family gene from finger millet.

Finger millet (*Eleusine coracana* (L.) Gaertn) is a drought hardy crop and widely grown in the arid areas of Africa and Asia and thrives well under disturbed soils, can grow in hot climates and survives severe water deficit and osmotic stress [36]. Comparative analysis for drought stress tolerance among different crop species showed that finger millet was highly drought tolerant with nearly 90% survivability similar to drought tolerant species peanut and horse gram. However, sunflower, beans and tomato were found to be highly susceptible to drought with only 20% survival rate [37]. Further, extensive screening of finger millet germplasms under drought conditions showed that GPU-28 belonged to tolerant group with a low drought susceptibility index (DSI) of 0.9–1 compared to susceptible genotype of DSI of 1.9. Also, in a study using induction response technique (34), GPU-28 had better acquired tolerance, hence this genotype was chosen for the present study. Due to this extreme hardiness, genes from finger millet would be a valuable resource to improve stress tolerance. From this context, a drought stress cDNA library of finger millet cv.GPU28 was constructed and several stress responsive transcription factors were identified [35, 38]. In the finger millet stress cDNA library a few *bHLH* ESTs were identified and the emphasis in this study was on cloning and characterization of stress responsive *EcbHLH57* from finger millet, a homolog of *OsbHLH057* and *AtILR3* in model plant tobacco. Overexpression of *EcbHLH57* in tobacco resulted in improved tolerance to diverse abiotic stresses.

## Materials and Methods

### Plant material and stress treatments

Finger millet genotype GPU-28 used in this study belongs to tolerant group of genotypes with a low drought susceptibility index (DSI) of 0.9 compared to susceptible genotypes with DSI of 1.9–2.0. Finger millet seedlings (var. GPU-28) were grown in small pots for one month and subjected to drought stress imposed gravimetrically [37]. The leaf material was collected at 100, 80, 60 and 40% field capacities (FC). For seedling level stress treatments, GPU-28 seeds were germinated in petridishes and 7-day-old seedlings were treated with 10 μM ABA, -12 bars polyethylene glycol (PEG)-6000, 300 mM NaCl and 10 μM MV. The treated seedlings were harvested at 12 and 24 h for expression analysis.

### Isolation of full-length sequence of *EcbHLH57* from finger millet

The partial length cDNA of *EcbHLH57* was isolated from finger millet stress cDNA library. The 5’ end was amplified using RNA Ligase Mediated Rapid Amplification of cDNA Ends (RLM-RACE) kit (Invitrogen Corporation, Carlsbad, CA, USA) according to manufacturer’s instructions. The full length sequence of 1053 bp was amplified using gene specific primers and the reaction was carried out under standard PCR conditions. Amplified product was cloned into T/A vector (pTZ57/R, MBI Fermentas) and sequenced. The protein sequence was analysed for potential motifs/domains using PLANTsP software tool (http://plantsp.genomics.purdue.edu/index.html). The protein sequences were multiple aligned with close homolog from different species using CLUSTALW multiple alignment tool (http://www.genome.jp/tools/clustalw/) and phylogenetic tree was deduced. These aligned sequences were submitted to SMS2 (http://www.bioinformatics.org/sms2/) to arrive at the conserved regions within the close homologs. The interaction of EcbHLH57 ortholog AtILR3 with other genes was studied using GeneMANIA tool (www.genemania.org)

#### Generation of transgenic tobacco overexpressing *EcbHLH57*

The pTZ57/R vector was digested with *XbaI* and *BamHI* to release *EcbHLH57* cDNA fragment and subcloned into cloning vector IM1.1RBCS. Subsequently PRBCS::*EcbHLH57*:Trbcs fragment was released by digesting IM1.1RBCS: *EcbHLH57* with *AscI* and *PacI* and cloned into pBINplus vector. The plasmid was then mobilized into *Agrobacterium tumefaciens* strain EHA105 by electroporation. Tobacco var. KST-19 was transformed by using *Agrobacterium* mediated tranformation method [39]. The transformants were selected on Murashige and Skoog (MS) agar medium [40] containing 100 mg/L kanamycin, 350 mg/L cefotaxime. The seeds obtained from T_0_ transformants were germinated on MS agar medium supplemented with 100 μg/mL kanamycin. Based on the phenotype of the seedlings under the selection pressure for 15-days, segregation ratio was calculated (S2 Table).

### Gene expression analysis

Total RNA was extracted according to the protocol described by Datta et al. [41] and first strand cDNA was synthesized by oligo (dT) primers using Molony Murine Leukaemia Virus reverse transcriptase (MMLV-RT; MBI Fermentas, Hanover, MD, USA) according to manufacturer’s instructions. The cDNA pool was used as a template to perform RT- PCR analysis. PCR conditions were 94°C for 2 min, 25 cycles of 94°C for 45 s, 52–58°C for 30 s, 72°C for 30 s and a final extension of 72°C for 10 min. The quantitative real-time RT-PCR was performed in a qPCR machine (RT-PCR; Opticon2, MJ research, USA) with the fluorescent dye SYBR-green (TAKARA SYBR-green qPCR Kit) following the manufacturer’s protocol. The conditions for the PCR were as follows: 95°C for 2 min, 25 cycles of denaturation at 94°C for 45 s, annealing for 30 sec (56°C and 58°C for genes and elongation factor, respectively), polymerization for 45 s (72°C) followed by plate reading at 72°C for 5 min, estimation of melting curve from 50°C to 95°C and incubation at 72°C for 4 min. The relative expression levels of the selected genes under a given stress condition was calculated using comparative threshold method. Elongation factor was used as internal control for tobacco samples and actin was used as internal control for finger millet samples to normalize qRT- PCR (For primer list See S1 Table). The reaction efficiency for each gene was calculated using software LinRegPCR [42] (S3 Table).

### Drought and salinity stress tolerance assays

For drought stress imposition, tobacco transgenic and wild type plants were grown in pots and when the plants reached 90 days, soil water status was gradually reduced by gravimetric approach. Gradual moisture stress was imposed by weighing the pots and the loss of water in the pots was replenished with required amount of water to arrive at the desired field capacity (FC) of soil. At the end of stress period when pots reached the required FC (60 and 40%), they were maintained in that particular FC for about 3 days [37]. One set of plants was maintained at 100% FC. Gas exchange parameters such as photosynthetic rate (μmolm^−2^s^−1^) and stomatal conductance (mmol^−2^s^−1^) were measured using a portable photosynthetic system (LICOR 6400, USA). The plants were assessed for dry matter accumulation by measuring the stem, leaves and root biomass.

30-day-old transgenic and wild type plants were subjected to drought stress by withholding water for 1 week and analysed for relative water content, electrolyte leakage, chlorophyll content and MDA levels.

Salinity stress was imposed on 30-day-old transgenic and wild type plants grown hydroponically by supplementing 300 mM NaCl for 7 days. At the end of stress period, the shoot and root fresh weight of plants, electrolyte leakage and chlorophyll content was recorded. For long-term salinity stress, 90-day-old pot grown plants were treated with 100 mM NaCl for 25 days and later grown upto maturity and yield parameters were recorded.

### Seedling level stress imposition

The germinated seeds of transgenic and wild type were placed on half MS agar medium supplemented with 100 mM NaCl. The seedling growth was observed 7 days after stress treatment. The fresh weight, root length and cotyledon greening of seedlings were recorded. Photographs were taken at the end of the stress period.

### Estimation of total chlorophyll content

Chlorophyll was extracted from 200 mg of leaf tissue in acetone:DMSO (1:1) and the supernatant was made up to a known volume. The absorbance was recorded at 663 nm and 645 nm using UV-visible spectrophotometer (UV 2450, Shimadzu Corporation, Kyoto, Japan). Total chlorophyll was estimated by using the following formula and expressed as mg/g FW [43].

$$\text{CHLa }=\;(12.7\;({\text{A}}_{663})-\;2.69\;({\text{A}}_{645})\;\times \text{ V})\;/\;(\text{W }\times \;1000)$$

$$\text{CHLb }=\;(22.9\;({\text{A}}_{645})-\;4.68\;({\text{A}}_{663})\;\times \text{ V})\;/\;(\text{W }\times \;1000)$$

TCHL (mg/g FW) = CHLa + CHLb 

Where, CHL = chlorophyll, V = volume, W = weight and TCHL = Total chlorophyll

### Electrolyte leakage assay

Leaf discs from 30-day-old transgenic and wild type plants subjected to NaCl treatment and drought stress were analyzed for membrane damage by electrolyte leakage assay using conductivity meter (Elico-India, CM183, EC-TDS analyser) [37]. The leaf discs were transferred to deionised water and incubated for 2 h and initial electrical conductivity (EC) was recorded. The samples were then boiled for 15 min and final EC of the resultant solution was recorded. The electrolyte leakage was calculated using the following formula: EC (%) = (IEC/FEC) x 100 where, IEC = initial EC, FEC = final EC

### Estimation of relative water content

Leaf discs from 30-day-old transgenic and wild type plants subjected to drought stress were analysed for relative water content. Leaf discs were initially weighed to record the fresh weight. Later they were floated in deionized water and incubated for 5 h. The discs were blotted on filter paper to remove excess water and recorded the turgid weight. The leaf discs were oven-dried and recorded the dry weight. The relative water content was estimated using the formula: RWC (%) = ((FW−DW) / (TW−DW)) x 100 where FW = fresh weight, DW = dry weight, TW = Turgid weight.

### Excised leaf disc assays

Leaf discs were made from identical leaves of 3-month-old transgenic and wild type plants were placed on half MS medium containing -6 bars PEG 6000, 200 mM NaCl, and 2 μM Methyl viologen (MV) and incubated for 72 h. After stress period, leaf discs were analysed for total chlorophyll content as described previously [43]

### Estimation of H_2_O_2_ levels

The leaf discs treated with MV was extracted in phosphate buffer of pH 7.5 and 25 μl supernatant was taken and mixed with 275 μl of xylenol orange reagent. The reaction mix was incubated for 30 min at room temperature and absorbance was measured at 560 nm against blank (xylenol orange reagent) [44]. The xylenol orange reagent was prepared in 50 ml of distilled water containing 1ml of 50 mM ferrous ammonium sulphate in 2.5 M H_2_SO_4_ and 62.5 μl of 125 μM xylenol orange along with 0.9019 g sorbitol.

### Estimation of malondialdehyde (MDA) content

MDA content was estimated by using TBARS assay. About 0.5–1.0 g of tissue was homogenized in 5 ml of 5% (w/v) trichloroacetic acid and the homogenate was centrifuged at 12,000 g for 15 min at room temperature. The supernatant was mixed with an equal volume of thiobarbituric acid (0.5% in 20% (w/v) trichloroacetic acid), and the mixture was boiled for 25 min at 100°C, followed by centrifugation for 5 min at 7,500 g to clarify the solution. Absorbance of the supernatant was measured at 532 nm and corrected for nonspecific turbidity by subtracting the A600. MDA content in leaf tissue was calculated using standard graph [45].

### Analysis of stomatal characteristics

In order to observe the stomatal characters, leaves from same aged plants and from same relative position was selected. The epidermal impressions were taken by applying a thin layer of thermocole paste in xylene on abaxial and adaxial surface of leaves. The imprint was removed and placed on glass slide and observed under light microscope with 40X magnification. The stomatal number and epidermal cell number was counted and calculated the stomatal index. The stomatal index was calculated by dividing number of stomata with total number of cells (i,e epidermal cells and stomatal cells) and multiplied by 100.

### Statistical analysis

The data obtained in different experimental results was analysed using two-way analysis of variance (ANOVA) as per the procedure given by Fischer [46]. Data points with different lowercase letters indicate significant differences (P<0.05) between transgenic lines and wild type as determined by Duncan’s multiple range test.

## Results

### Expression analysis of *EcbHLH57* under different stress conditions in finger millet

**Fig 1:**
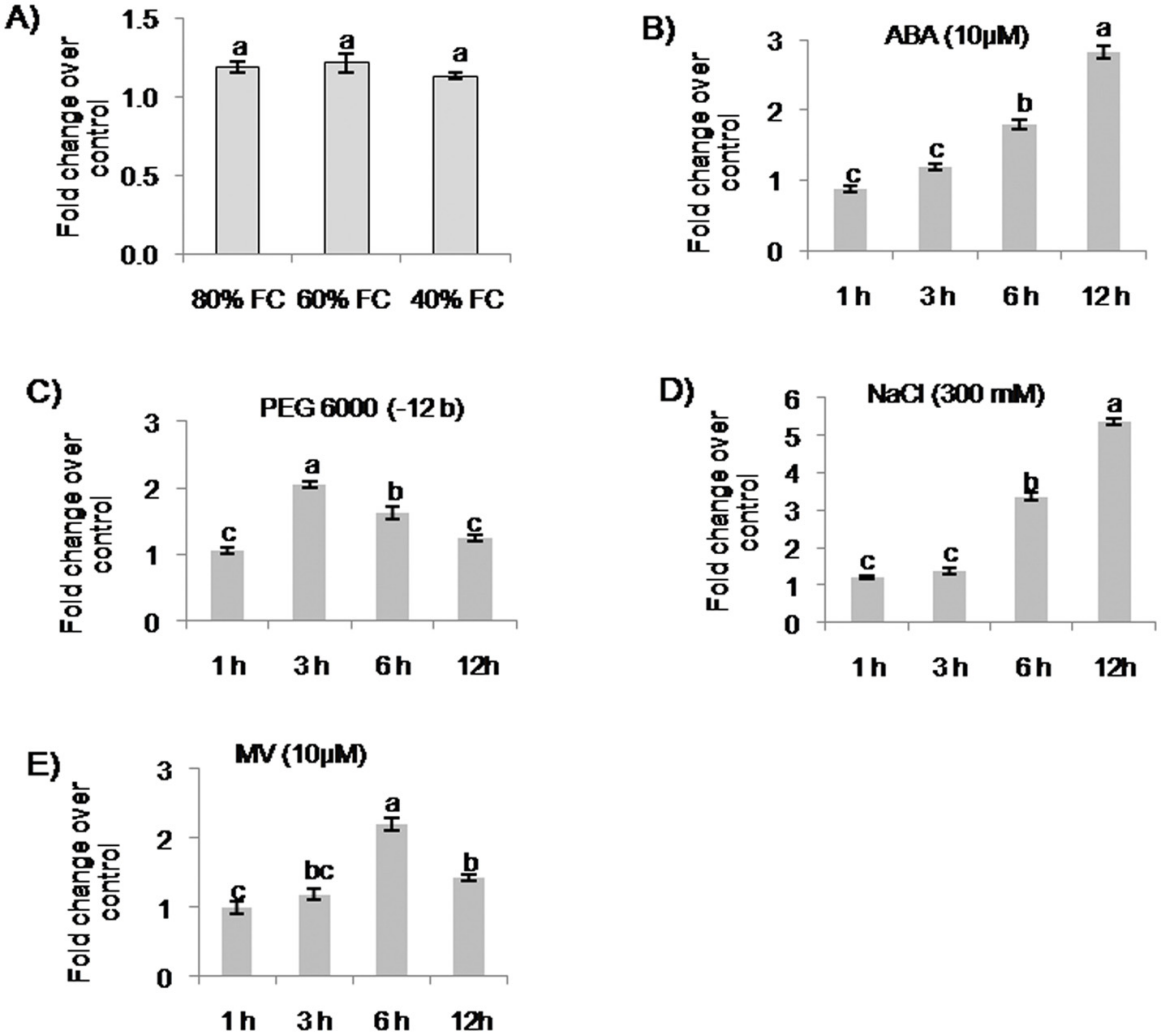
Expression analysis of *EcbHLH57* in finger millet under different stress conditions. A) Quantitative RT-PCR analysis of *EcbHLH57* in finger millet plants subjected to drought stress of 80%, 60%, 40% FC and in seedlings treated with B) ABA (10 μM), C) PEG 6000 (-12 bars), D) NaCl (300 mM) and E) MV (10 μM) at different time points. Total RNA was isolated and cDNA prepared was used for quantitative RT-PCR analysis. *Actin* was used as internal control. Data represent mean of three replications (n = 3) and bars indicate standard error of mean. The lowercase letters that are different indicate significant difference (Duncan’s multiple range test, P<0.05) between treatment conditions.

The *bHLH* EST obtained from finger millet stress cDNA library was characterized for its stress responsiveness through expression analysis under different stress stimuli such as drought, NaCl, PEG, ABA and MV treatments. Pot-grown finger millet plants were subjected to drought stress by gradually reducing the soil moisture content to 80, 60 and 40% FC through gravimetric method. Non-stressed plants were maintained at 100% FC. At the end of stress period, the plants showed a reduction in chlorophyll content and membrane integrity with decreasing field capacity (data not shown). The drought stressed leaves analyzed for *EcbHLH57* expression showed increase in transcript levels (1.3 fold) at 80 and 60% FC over water control (100% FC), however the levels were reduced at 40% FC but no significant differences were observed between treatments (Fig 1A). However the transcript levels under ABA at 12 h and PEG at 3 h that simulate the drought stress response were significantly higher (Fig 1B and 1C) in finger millet seedlings. Under NaCl treatment, the transcript levels showed a gradual increase with time points and peaking at 12 h (Fig 1D). The transcript levels were also induced under MV treatment with a peak at 6 h but reduced at 12 h (Fig 1E).

### Isolation and sequence analysis of *EcbHLH57*

The identified *bHLH* EST was found to be stress responsive and hence the full length cDNA was cloned from finger millet for further characterization. The partial length EST lacked the 5’ end with an intact 3’ end. The 5’ end was cloned by using RACE protocol. Later the full-length cDNA sequence of *bHLH* gene was isolated from finger millet. The sequence analysis indicated that the coding region was 771 bp starting from 100 bp to 870 bp, 5′ UTR from 1 to 99 bp and 3′ UTR from 871 to 1050 bp and poly-A signal from 1042–1047 bp encoding a 28.16 kDa protein of 256 amino acids (S1 Fig). The gene was designated as *EcbHLH57* based on its homology with *OsbHLH*057. EcbHLH57 (KP064138) shares 75% homology with rice homolog LOC_Os07g35870 (NP_001059905) (OsbHLH057; Li *et al*., 2006), 79% with *Brachypodium distachyon* ILR3-like (XP_003562971), 77% with ZmbHLH (XP_008651108), 55% with VvILR3-like (XP_002274829), 55% with MtILR3 (XP_003607701), 53% with AtILR3 (AtbHLH105) (NP_200279), and 53% with GmILR3-like isoform 1(XP_003527314). The amino acid sequence alignment of close orthologs of EcBHLH57 revealed a conserved bHLH domain 89–143 aa followed by a highly conserved leucine repeats similar to *Arabidopsis* bHLH members of group IV (S2A Fig). Phylogenetic analysis based on amino acid sequence of EcbHLH57 and bHLH from other close orthologs showed that it is clustered with sorghum and maize bHLH and is evolutionarily clustered to ILR3 group from different species (S2B Fig). The EcbHLH57 ortholog from *Arabidopsis* AtILR3 was used to predict interacting proteins using GENE MANIA tool which showed physical interaction with PYE, bHLH115, bHLH104 and BRUTUS (BTS) proteins suggesting that bHLH forms heterodimers. The co-expression network showed many genes such as bHLH TFs, PYL 5 (ABA receptor), HVA 22 like proteins (late embryogenesis abundant-LEA protein) were expressed along with AtILR3 (S3 Fig).

### Development of tobacco transgenic plants expressing *EcbHLH57* and phenotypic analysis

**Fig 2:**
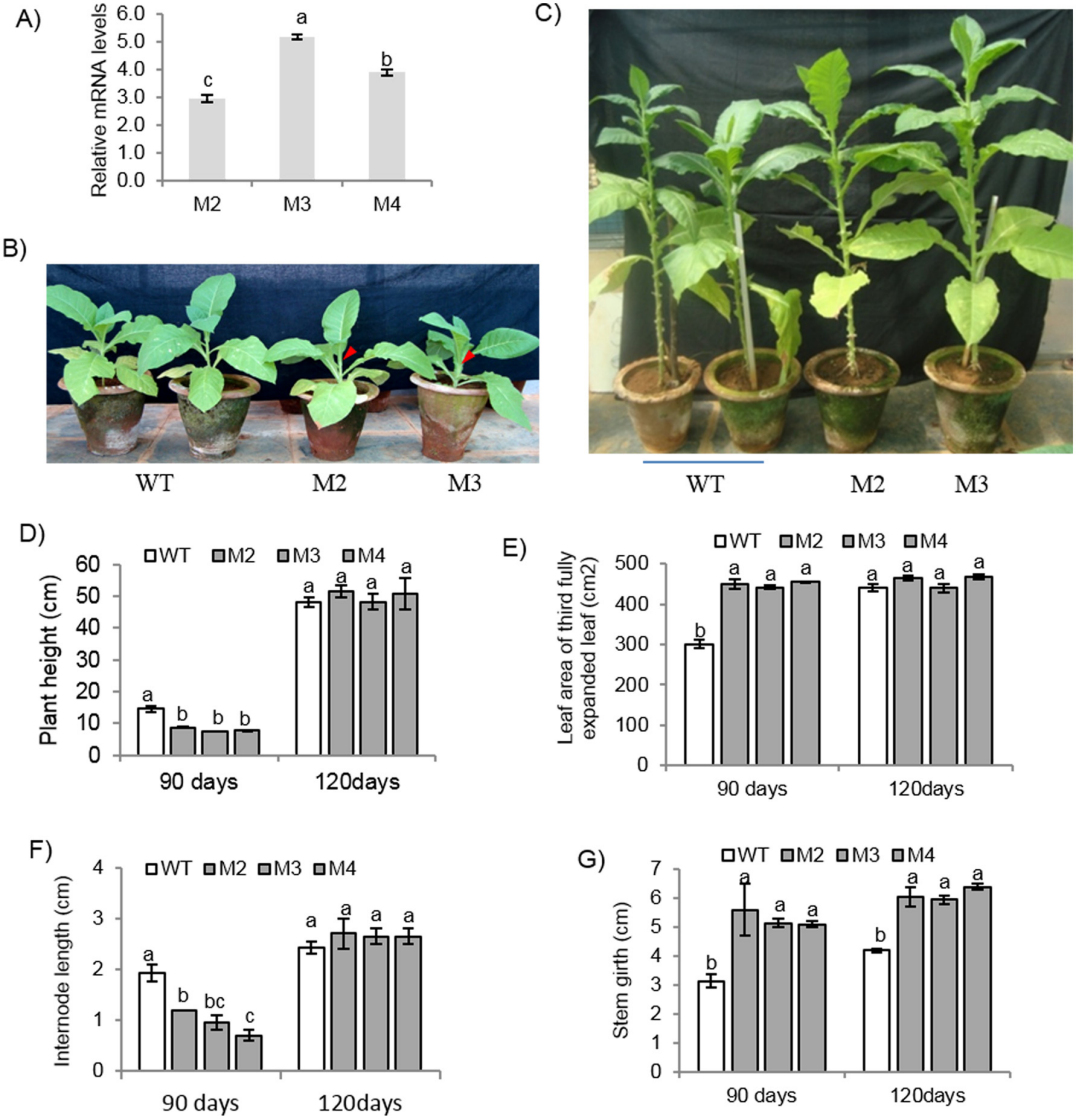
Expression and phenotypic analysis of *EcbHLH57* expressing tobacco transgenic and wild type plants. A) qRT-PCR analysis of tobacco transgenic plants overexpressing *EcbHLH57*. Photograph showing phenotype of wild type and transgenic plants at B) 90 days and C) 120 days after germination. Histogram showing D) Plant height and E) Leaf area of third fully expanded leaf, F) Internode length and G) Stem girth of transgenic and wild type plants. Arrows indicate distance between two internodes. Data represent mean of three replications (n = 3) and bars indicate standard error of mean. The lowercase letters that are different indicate significant difference (Duncan’s multiple range test, P<0.05) between transgenic and wild type plants. WT-wild type, M2, M3 and M4- Transgenic plants.

**Fig 3:**
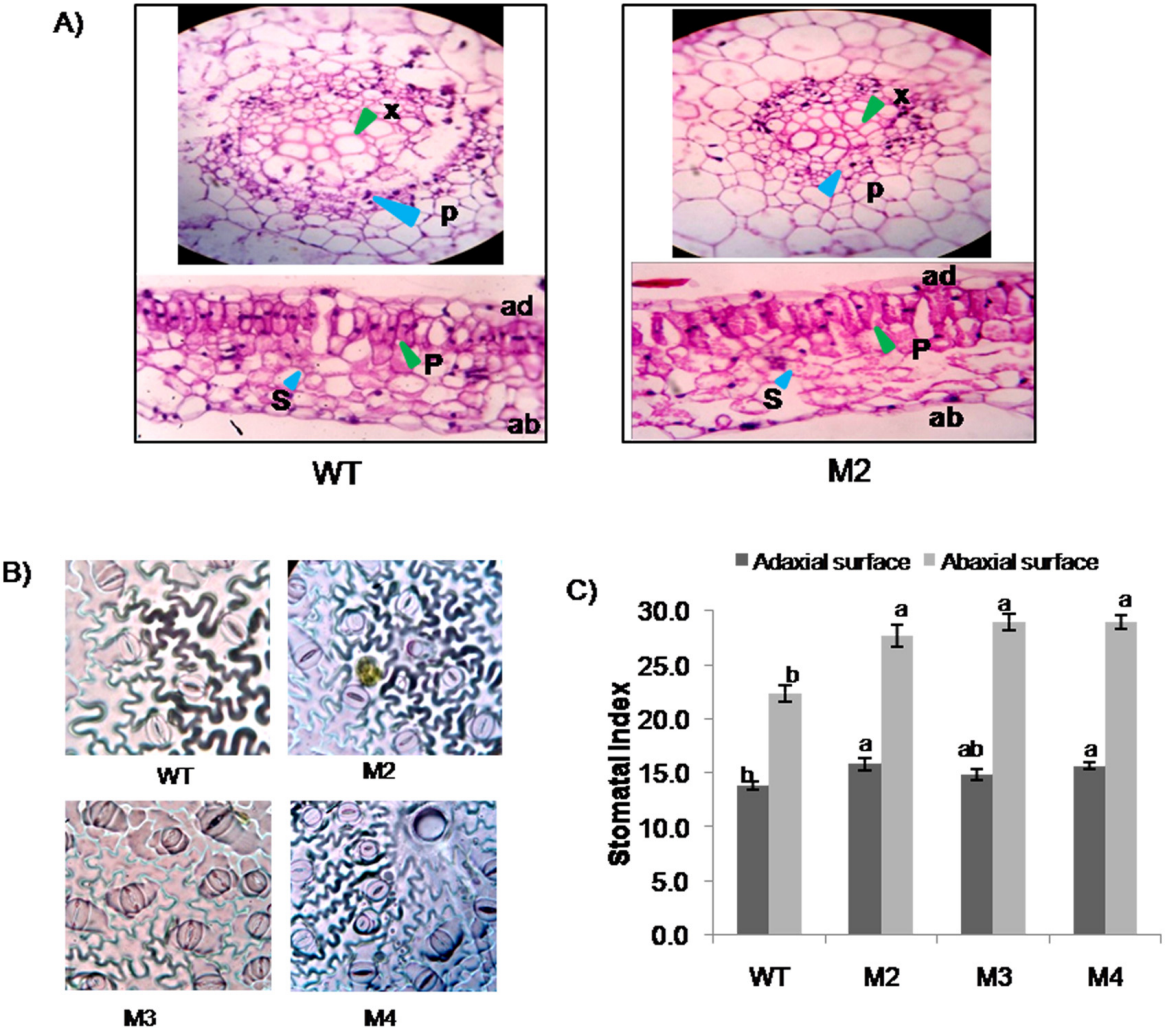
Leaf anatomical study and microscopic view of epidermal impression of transgenic and wild type plants (40X magnification). A) Leaf cross-sections observed under microscope. For histological studies, the top third leaf of transgenic and wild type plants was taken and microtome sections were made. Staining was done using eosin (stains nucleus) and hematoxylin (stains cytoplasm). B) Photograph showing abaxial surface of leaves from transgenic and wild type plants. C) Graph depicting stomatal index of transgenic and wild type plants. Data represent mean of three replications (n = 3) and bars indicate standard error of mean. The lowercase letters that are different indicate significant difference (Duncan’s multiple range test, P<0.05) between transgenic and wild type plants. x- xylem, p-phloem, S-spongy parenchyma, P-palisade parenchyma, ad-Adaxial, ab- abaxial.

In order to investigate the function of *EcbHLH57*, transgenic tobacco plants were developed overexpressing *EcbHLH57* cDNA driven by Ribulose-1,5-bisphosphate carboxylase small subunit (RBCS) promoter. The T_1_ seeds of twenty four independent transgenic lines were screened on kanamycin containing media and three lines M2, M3 and M4 segregating in the ratio of 3:1 were selected (S2 Table) and advanced to T_2_ and subsequently to T_3_ generation. These transgenic plants showed higher transcript levels and differential expression pattern of *EcBHLH57* (Fig 2A). The *EcbHLH57* expressing transgenic plants showed phenotypic variations during initial stages of growth under normal conditions (Fig 2B). The transgenic plants showed an initial reduction in plant height at 90 days of growth with reduced internode length resulting in short stature of plants (Fig 2D and 2F). However, the leaf area of third fully expanded leaf was significantly higher at 90 days. The stem girth was also higher in these transgenic plants and was maintained throughout the growth period (Fig 2E and 2G). The growth was recovered at later stages as evidenced at 120 days and the transgenic plants were on par with wild type plants. Since the transgenic plants showed growth differences, leaf anatomy and stomatal characters were studied. The transgenic plants did not show any difference in the alignment and size of palisade parenchyma and spongy parenchyma cells compared to wild type plants. However, the size of xylem and phloem vessels was reduced than wild type plants (Fig 3A). The leaf epidermal impressions showed that, the transgenic plants had higher number of stomata and epidermal cells with increased stomatal index on the adaxial and abaxial surface compared to wild type plants (Fig 3B and 3C). However the size of epidermal cells was relatively smaller than that of wild type plants. Further to assess whether the differences was due to any cell cycle related genes, qRT-PCR was carried for few cell cycle genes like *cell division cycle 6*, *45*, *origin recognition complex* (*ORC1B*), cyclin B2.2 (cyb2.2), *Mini*-*chromosome maintenance proteins* (*MCM 2* and *MCM10*). The expression of these genes did not alter significantly (S4 Fig).

### Response of *EcbHLH57* expressing transgenic plants to NaCl induced salinity stress

**Fig 4:**
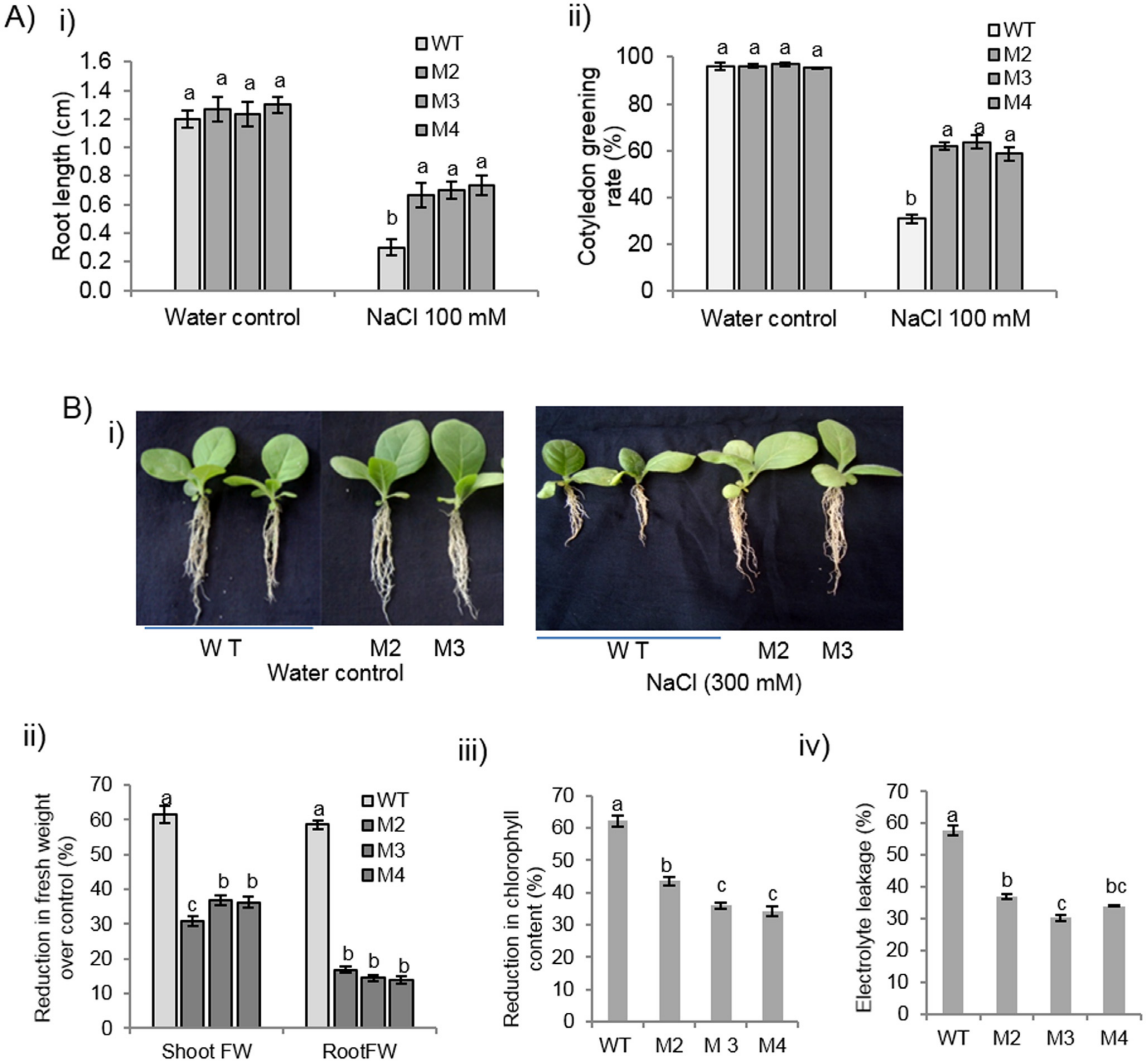
Response of *EcbHLH57* expressing transgenic plants for salinity stress tolerance. A) Root length and cotyledon greening rate of *EcbHLH57* expressing transgenic and wild type seedlings under 100mM NaCl treatment. Graph depicting i) Root length and ii) Cotyledon greening rate of transgenic and wild type plants. B) 30-day-old plants were treated with 300 mM NaCl for 1 week. i) Root growth comparison of transgenic and wild type plants. ii) Per cent reduction in shoot and root fresh weight of plants over water control iii) Percent reduction in chlorophyll content. iv) Electrolyte leakage under NaCl treatment. Data represent mean of three replications (n = 3) and bars indicate standard error. The lowercase letters that are different indicate significant difference (Duncan’s multiple range test, P<0.05) between transgenic and wild type plants exposed to same treatment. WT- wild type. M2, M3 and M4- transgenic lines expressing *EcbHLH57*.

The response of tobacco transgenic plants to salinity stress was studied by growing the germinated seedlings on half MS medium supplemented with 100 mM NaCl for 7 days. The transgenic plants maintained significantly higher growth on NaCl medium and accumulated 40% higher fresh weight than wild type seedlings (S5 Fig). The transgenic seedlings showed greener cotyledonary leaves and better root growth than the wild type seedlings which showed reduced growth (Fig 4A).

Further to assess the tolerance at whole plant level, the transgenic and wild type plants were grown for 30 days hydroponically in Hoagland’s solution. Subsequently these plants were transferred to nutrient media containing 300 mM NaCl solution and maintained for 7 days. Transgenic plants showed improved tolerance with less reduction in shoot and root fresh weight of 35 and 17% respectively compared to wild type with 60% reduction (Fig 4Bii). The transgenic plants showed better root growth but the wild type plants were severely affected with fewer roots. The membrane damage was significantly less in transgenic plants with 33% electrolyte leakage as compared to 57% in wild type plants. The transgenic plants also maintained higher chlorophyll content with 38% reduction under salinity stress, however the wild type plants showed a significantly high reduction in chlorophyll content of 62% (Fig 4Biii and 4Biv).

Further long term salinity stress response was assessed in 90-day-old pot-grown plants. The transgenic plants were less affected than wild type plants and accumulated higher biomass, seed weight per pod and higher number of pods per plant. But the wild type plants showed a reduction in the yield parameters (S4 Table).

### Transgenic plants showed tolerance to PEG, NaCl and MV induced stress

**Fig 5:**
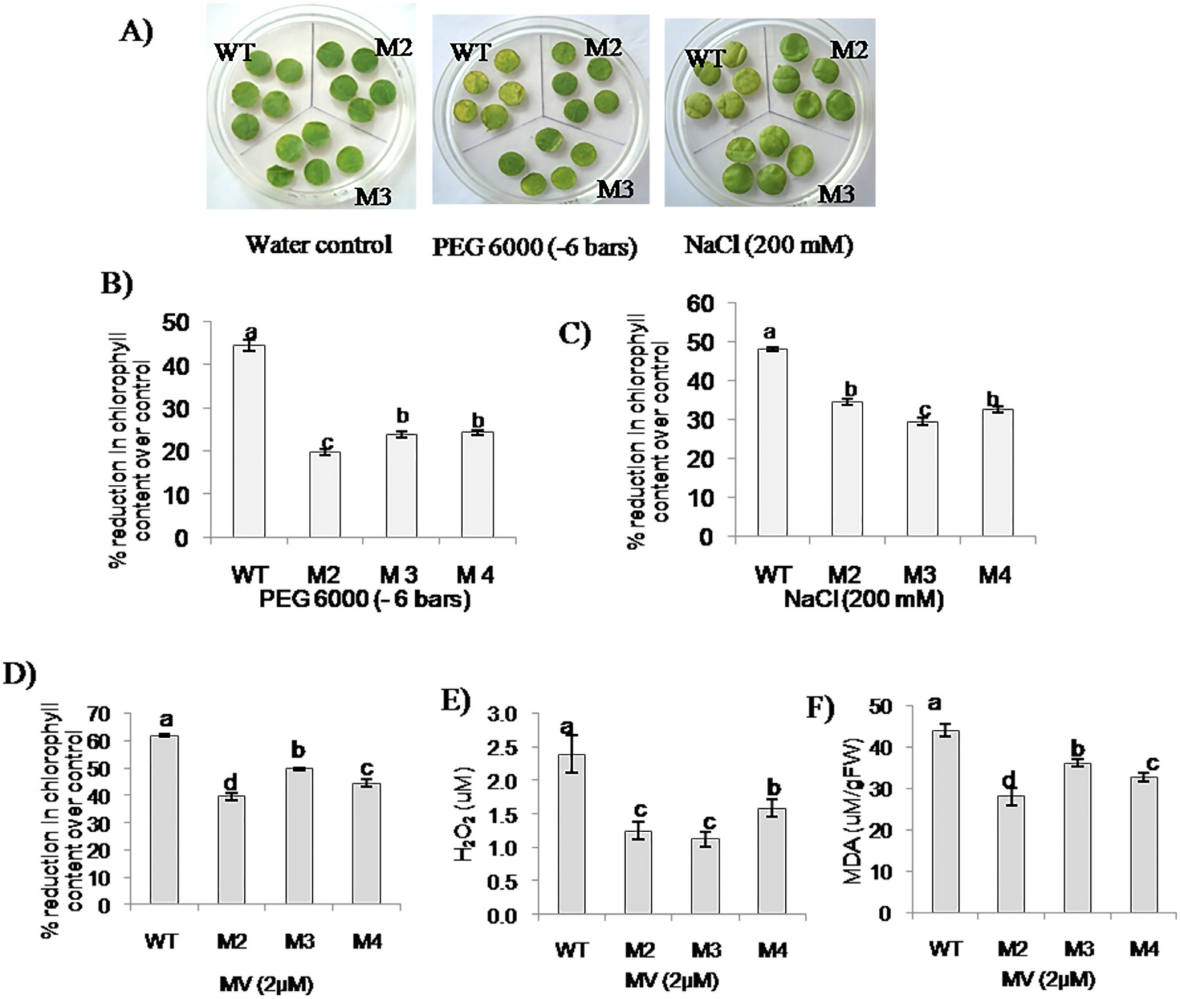
Response of *EcbHLH57* expressing transgenic plants to PEG, NaCl and MV induced stress. Leaf discs from wild type and transgenic plants were placed on half MS medium supplemented with PEG 6000 (-6 bars), NaCl (200 mM) and MV (2 μM). A) Photograph taken 72 h after exposure to treatment. Leaf discs were analyzed for per cent reduction in chlorophyll content over water control under, B) PEG, C) NaCl and D) MV treatments. Analysis of, E) H_2_O_2_ content using xylenol orange assay and, F) MDA content using TBARS assay in MV treated leaf discs. Data represent mean of three replications (n = 3) and bars indicate standard error. The lowercase letters that are different indicate significant difference (Duncan’s multiple range test, P<0.05) between transgenic and wild type plants exposed to same treatment. WT- wild type. M2, M3 and M4- transgenic lines expressing *EcbHLH57*.

To study the response of transgenic plants to different abiotic stress inducers, leaf disc assays was carried out using different stress inducing compounds such as PEG 6000 (-6 bars), NaCl (200 mM) and MV (2 μM). The transgenic plants showed 21 to 25% reduction in chlorophyll content on PEG 6000 and 30 to 35% in NaCl treatments respectively over water control, however wild type plants showed 45% reduction under both stress treatments (Fig 5B and 5C) indicating an improved tolerance in *EcbHLH57* expressing transgenic plants.

Further, the leaf discs from transgenic plants placed on medium containing MV showed significant tolerance to oxidative stress with less reduction in chlorophyll content (Fig 5D). The wild type plants showed 60% reduction in chlorophyll content but the transgenic plants maintained higher chlorophyll content with 45% reduction over water control. The MV treated leaf discs of transgenic plants accumulated reduced levels of both H_2_O_2_ and MDA products. Transgenic plants showed 1.1 to 1.5 μM of H_2_O_2_ and 28–35 μM of MDA content than wild type plants which accumulated 2.4 μM of H_2_O_2_ and 45 μM of MDA (Fig 5E and 5F). The data demonstrates enhanced tolerance of transgenic plants to oxidative stress.

### Response of *EcbHLH57* expressing transgenic plants to drought stress at seedling level

The drought stress response of transgenic plants was assessed in 30-day-old plants by withholding irrigation water for 1 week. At the end of stress period, the wild type plants showed severe wilting symptoms with 45% relative water content with a higher reduction in chlorophyll content (51%) and higher membrane damage (46%). However the transgenic plants showed improved tolerance to drought stress and maintained higher relative water content of 64%. The transgenic plants also showed less reduction in chlorophyll content (26%) with reduced electrolyte leakage (21%) and MDA content (S6 Fig).

### Effect of gradual moisture stress on *EcbHLH57* overexpressing tobacco transgenic plants

**Fig 6:**
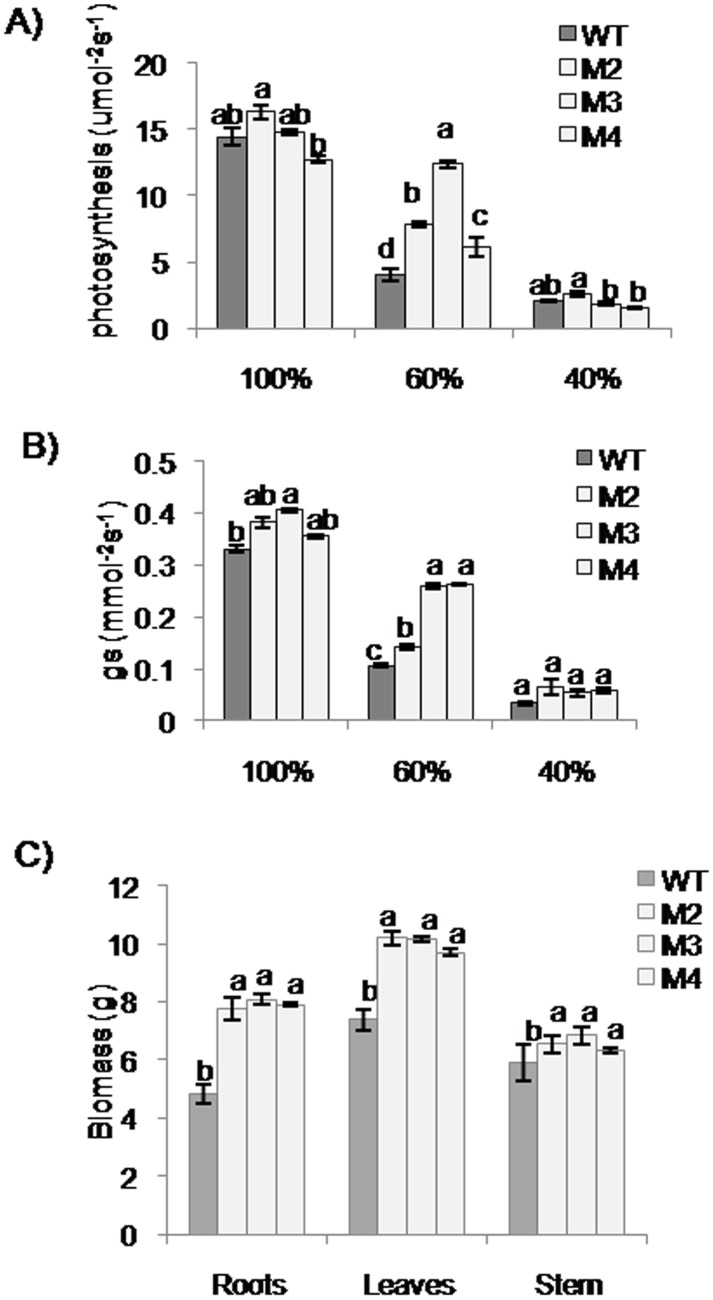
Effect of gradual moisture stress on photosynthetic efficiency in *EcbHLH57* expressing transgenic plants. Moisture stress was imposed by gradual reduction of soil moisture status to 60% and 40% FC. 100% FC was maintained as control. Photosynthetic measurements were made at the end of the stress period using IRGA (LiCOR, USA). A) The photosynthesis. B) Stomatal conductance and C) Comparison of dry weight of transgenic and wild type plant at the end of stress period. Data represent mean of three replications (n = 3) and bars indicate standard error. The lowercase letters that are different indicate significant difference (Duncan’s multiple range test, P<0.05) between transgenic and wild type plants exposed to same treatment. WT- wild type. M2, M3 and M4- transgenic lines expressing *EcbHLH57*.

To study the long term effect of drought stress on transgenic plants, 90-day-old pot-grown transgenic and wild type plants grown under well-watered conditions were subjected to drought stress through gravimetric approach. At the beginning of stress (100%) and when soil moisture reached 60 and 40% FC, photosynthetic gas exchange parameters were recorded using IRGA (LiCOR, USA). The carbon assimilation at 100% FC did not change significantly between transgenic and wild type plants. Under 60% FC stress conditions, the transgenic plants showed significantly higher rate of photosynthetic carbon assimilation and stomatal conductance compared to wild type plants. But under severe stress of 40% FC, the photosynthetic efficiency of transgenic tobacco plants was on par with that of wild type plants (Fig 6A and 6B). However the transgenic plants accumulated significantly higher shoot and root biomass compared to wild type plants at the end of stress period (40% FC) (Fig 6C).

### *EcbHLH57* transcription factor enhanced the expression of stress responsive genes in transgenic tobacco plants under drought stress

**Fig 7:**
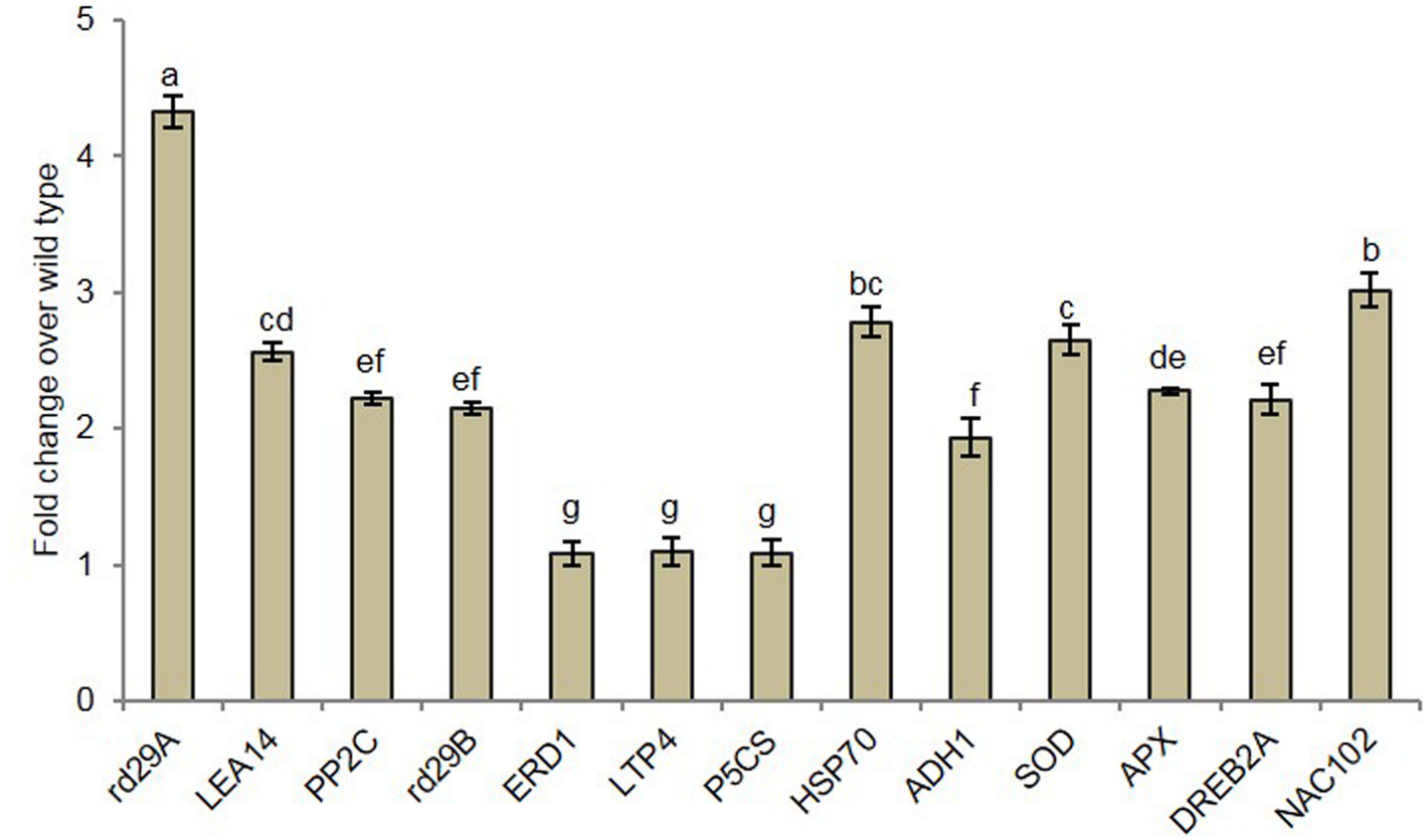
Expression analysis of downstream genes under drought stress in *EcbHLH57* transgenic plants. Quantitative RT-PCR analysis of a few downstream genes in transgenic and wild type plants under drought stress conditions. *Elf-* elongation factor was used as internal control. Data represent mean of three replications (n = 3) and bars indicate standard error. The lowercase letters that are different indicate significant difference (Duncan’s multiple range test, P<0.05) between means of analyzed genes.

To study the genes regulated by EcbHLH57, the expression levels of downstream stress responsive genes such as *LEA14*, *PP2C*, *ERD1*, *LTP4*, *P5CS*, *rd29A*, *rd29B*, *HSP70*, *ADH1*, *SOD*, *APX* and TFs *DREB2A* and *NAC102* were analysed under drought stress. Analysis of promoter sequences of these genes indicated that the genes *LEA14*, *LTP4 P5CS*, *ADH1*, *APX*, *DREB2A* and *NAC102* contained G-box cis-element, however other genes did not show the presence of this cis-element. Quantitative RT-PCR analysis indicated that the transcript levels of rd29A was increased by 4- fold in transgenic plants upon drought stress. The expression of other genes such as *LEA14*, *PP2C*, *rd29B*, *ADH1*, *APX* and *DREB2A* was also increased by 2-fold and the genes *HSP70*, *SOD* and *NAC102* showed ~3-fold increase over wild type. However the expression of *LTP4* and *P5CS* was itself very high in wild type and the expression in transgenic plants was on par with wild type (Fig 7).

## Discussion

The plant bHLH TFs constitute one of the largest families, however, only a few members have been functionally characterized for stress response. In *Arabidopsis AtMYC2*, *AtbHLH17*, *AtbHLH92* and *AtICE1* TFs have been shown to be induced by ABA osmotic, salinity and cold stress respectively [4, 47, 22, 23]. *OrbHLH001* and *OrbHLH2* from wild rice were induced by NaCl [32, 33]. Several other bHLH TFs such as *OsbHLH1* [24], *TaICE41*, *TaICE87* [48], *MdCIbHLH1* [25] and *PtrbHLH* [49] were induced upon cold stress. [22]. In our study, the expression of a *bHLH* TF (*EcbHLH57*) identified from finger millet stress cDNA library was upregulated under different stress treatments such as ABA, NaCl, PEG, MV and drought stress (Fig 1). Although *EcbHLH57* showed a marginal increase in transcript levels under drought stress, ABA and PEG treatments which simulate drought stress induced the accumulation of transcripts at early time points. ABA triggers drought signaling cascade and induces the expression of several genes involved in drought stress [50]. In many crop plants, exogenous application of ABA imparted resistance to drought [51, 52, 53]. Polyethylene glycol acts as a non-penetrating osmotic agent and increases solute potential and reduces water absorption by roots thereby induces drought stress [54, 55]. The expression analysis suggests that *EcbHLH57* is an early stress inducible gene and hence the transcript levels were not increased drastically under long-term stress induction during 80, 60 and 40% FCs. The e-northern analysis of *Arabidopsis* homolog *AtbHLH105* indicated that the transcripts were downregulated under salinity, drought and oxidative stresses. Similarly, the expression level of rice homolog *OsbHLH57* was also downregulated under drought and cold stress, but was significantly upregulated under salinity stress (data not shown). On the contrary, the increased expression of *EcbHLH57* under NaCl, ABA, PEG and MV substantiates its stress responsiveness in finger millet.

The identified *EcbHLH57* contains a *bHLH* domain followed by a leucine zipper (ZIP) motif. Phylogenetic analysis of EcbHLH57 with other bHLH proteins showed that it is closely related to ILR3 homologs in different species (S2B Fig). In *Arabidopsis*, bHLH proteins have been grouped into 12 families and ILR3 belongs to group IVc which have a highly conserved leucine zipper (ZIP) motif immediately after the bHLH motif [56]. EcbHLH57 has highest sequence similarity with BdILR3. The bHLH proteins from animal system belonging to group B also have a bHLH-Leu zipper motif. These include Max, Myc, MITF, SREBP, and USF which are involved in diverse processes [57, 58, 59]. It has been reported that, this ZIP motif stabilizes the dimerization of proteins [60, 61]. The binding specificity of these bHLH proteins has been identified as G-box motif (CACGTG). The prediction of interactive partners of AtILR3 which is a close homolog of EcbHLH57 showed that it physically interacts with other bHLH proteins such as PYE, bHLH115, bHLH104 to form functional heterodimers (S3A Fig). The co-expression network shows that many bHLH family genes are co-expressed with AtILR3. Apart from this, two genes PYL5, an ABA receptor and HVA22-like protein (AT5G42560) which is an ABA induced LEA protein [62] were also co-expressed with AtILR3 (S3B Fig). From this context, it can be speculated that EcbHLH57 may have a role in ABA mediated signaling and hence in imparting stress tolerance. The *EcbHLH57* overexpressing transgenic tobacco plants showed growth variations during initial stages under normal conditions. Although there was a reduction in the internode length, the transgenic plants showed increased leaf area. Analysis of leaf anatomy did not show any significant changes but reduced size of xylem and phloem vessels were observed. The expression of cell-cycle regulating genes was also unaltered in the transgenic plants (S4 Fig). However, the transgenic plants showed increased stomatal number and stomatal index on both adaxial and abaxial surfaces (Fig 3C) indicating that *EcbHLH57* may act as a regulator of stomatal development. The stomatal development involving cell fate transitions is regulated by several bHLH transcription factors, SPEECHLESS (SPCH), MUTE and FAMA [63] are expressed in different cell types such as young epidermal cells, late meristemoids and guard mother cells (GMC) and in immature guard cells respectively. Further other bHLH TFs such as Inducer of CBF Expression1/SCREAM (ICE1/SCRM) and SCRM2 form heterodimers with SPCH, MUTE and FAMA and promote stomatal fate transitions [64]. Therefore, *EcbHLH57* being a bHLH group of TF may have a role in regulating the levels of these TFs or by interacting with these resulting in increased stomatal number. However, further studies are needed in this direction to identify targets of EcbHLH57 and its role in stomatal development.

The *EcbHLH57* overexpressing tobacco transgenic plants showed improved tolerance to different abiotic stress treatments. The increased root growth, reduced electrolyte leakage and higher chlorophyll content of transgenic plants under salinity stress indicate enhanced tolerance (Fig 4). Chlorophyll content is highly sensitive to salt stress and its degradation in response to salinity has been reported [65, 66]. Similarly, electrolyte leakage is an indicator of damage caused by reactive oxygen species (ROS) induced by salt stress [67]. Hence the maintenance of these parameters in transgenic plants under salinity stress indicate their stress tolerance. The transgenic plants also showed higher plant height and biomass under long-term NaCl treatment and showed significantly higher seed weight/pod and pod number than wild type plants (S4 Table). Many other *bHLH* family genes have also been reported to improve salinity stress tolerance [22, 32, 33, 47]. The transgenic plants showed tolerance to simulated PEG, NaCl and MV stress treatments. Lower levels of H_2_O_2_ and MDA in MV treated transgenic plants suggest enhanced ROS scavenging machinery. Chlorophyll content is an indicator of photosynthetic capacity of plants [68]. Drought stress induces the production of ROS leading to lipid peroxidation which causes damage to the membrane system, photosynthetic pigments thus affecting photosynthesis [69, 70]. Higher levels of chlorophyll content, reduced membrane damage and less accumulation of MDA levels in *EcbHLH57* expressing transgenic plants subjected to drought stress suggests improved stress tolerance (S6 Fig). The plants also showed higher photosynthetic rate under drought stress due to better maintenance of photosynthetic pigments leading to increased accumulation of biomass (Fig 6). It has been well documented that higher photosynthesis contributes for improved leaf area and growth [71] as a result the transgenic plants showed improved root and shoot biomass under stress conditions (Fig 6C). The transgenic plants showed improved tolerance to different stress conditions, however the observed phenotypic and stress responses did not correlate with the differences in transgene expression levels in different transgenic lines. This could be due to the accumulated levels of the expressed protein might be sufficient to induce the observed phenotype. Recently Meng et al. [72] also reported that transgenic *Arabidopsis* plants expressing *35S*:*CLE14-CLE1* at lower levels could induce phenotypic severity and that variation in transgene levels did not correlate with phenotypic severity.

The *EcbHLH57* showed tolerance to multiple stresses and this phenomenon has been reported in many transgenic plants overexpressing TFs through upregulation of several stress responsive genes (4, 5, 6, 7, 35, 48). *DREB1A/CBF3* overexpressing transgenic plants showed improved tolerance to drought, salinity and low temperature by upregulating expression of dehydrin group of genes like *LEA*, *ERD10*, *COR15A* and *rd29A* [73]. *OrbHLH2* transgenic plants showed tolerance to salinity and osmotic stress through upregulation of stress responsive genes such as *rd29A*, *COR15A* and *KIN1* [34]. The *EcbHLH57* overexpressing transgenic tobacco plants also showed the upregulation of stress responsive genes like *LEA14*, *rd29A rd29B*, *HSP70*, *ADH1*, *SOD* and *APX* (Fig 7). The co-expression network analysis shows the expression of HVA-22 like protein (LEA) supporting the upregulation of LEA in these tobacco transgenic plants. The dehydrin family proteins are crucial in maintaining protein structure and stability under stress conditions. Under water stress conditions, the amphipatic α-helices of dehydrins interact with the partly dehydrated surfaces of proteins and stabilize their structure [74]. The increased transcript levels of dehydrins such as *LEA14*, *rd29A* and *rd29B* in *EcbHLH57* transgenic plants under stress conditions suggest better stability of macromolecules. Further, HSP70, a chaperone involved in refolding of unfolded proteins to maintain protein quality [75] also showed higher expression in transgenic plants. Drought stress also leads to the production of ROS [70] and higher transcript levels of *SOD*, *APX* and *ADH1* genes in transgenic plants indicates better detoxification of ROS species and hence reduced accumulation of MDA content. Overexpression of *SOD* from *Arachis hypogea* in tobacco resulted in improved tolerance to salinity and drought stress [76]. Similarly *ADH1* gene involoved in detoxification of several reactive carbonyl compounds and its overexpression in *Arabidopsis* conferred tolerance to abiotic stress by reducing ROS induced lipid peroxidation [77]. The higher expression of TFs such as *DREB2A* and *NAC102* in transgenic also suggests indirect upregulation of several other stress responsive genes which did not have bHLH binding motif. Earlier in *Arabidopsis* we showed that the target genes which do not have specific binding cis-elements of the overexpressed TFs may be upregulated by other TFs [47]. Further, the transcript level of *PP2C* was also upregulated in *EcbHLH57* transgenic plants. In response to stress, under high ABA levels, PP2C interacts with ABA receptor to activate SRK2D/E/I which upregulates several downstream genes such as LEA proteins [78]. The *Arabidopsis* co-expression network analysis also indicated the association of AtILR3 with ABA signalling pathway genes. The upregulation of *PP2C* in *EcbHLH57* transgenic plants indicates its positive role in improving stress tolerance via ABA signalling. Although *EcbHLH57* showed a marginal increase in transcript levels under drought, *EcbHLH57* being an early stress inducible gene, its constitutive overexpression in tobacco resulted in upregulation of several stress responsive target genes under drought stress such as *LEA14*, *rd29A* which help in stabilizing proteins, *SOD*, *APX*, *ADH1* scavenge stress induced ROS thereby aid in the maintenance of cell metabolic activities. Hence the physiological parameters like electrolyte leakage, chlorophyll content, photosynthesis, stomatal conductance and relative water content were maintained in the transgenic plants resulting in improved tolerance to abiotic stress conditions.

## Supporting Information

S1 Fig Full-length nucleotide sequence of *EcbHLH57*. The length of nucleotide sequence is 1050 bp, sequence in brown- 5’UTR (1–99 bp); black- coding region (771 bp, 100–870 bp); green- 3’UTR (871–1050 bp); blue- polyA signal
(PDF)

S2 Fig Comparison of EcbHLH57 with its close homologs. A) Phylogenetic analysis of EcbHLH57 with closely related members of bHLH family. Multiple sequence alignment was performed using ClustalW and phylogenetic tree was constructed. B) Alignment of deduced amino acid sequences of bHLH genes. box- bHLH domain, *- leucine residues
(PDF)

S3 Fig Interaction network for EcbHLH57 ortholog in *Arabidopsis thaliana* (AtILR3). A) Physical interaction. B) Co-expression
(PDF)

S4 Fig Expression analysis of cell-cycle regulating genes in *EcbHLH57* expressing transgenic plants under normal growth conditions. Graph depicting qRT-PCR analysis of cell-cycle regulating genes.
(PDF)

S5 Fig Salinity stress response of *EcbHLH57* expressing transgenic plants. Germinated seeds of transgenic and wild type plants were placed on half MS media supplemented with 100 mM NaCl. i) Growth comparison of transgenic and wild type seedlings on 100 mM NaCl induced stress. ii) Fresh weight of seedlings after stress period.
(PDF)

S6 Fig Response of *EcbHLH57* expressing tobacco transgenic to drought stress. 30-day-old plants were subjected to drought stress by withholding water for 1 week. A) Phenotype of plants under drought stress. B) Percent reduction in chlorophyll content C) Relative water content (%) D) Electrolyte leakage and E) MDA content in drought stressed plants.
(PDF)

S1 Table List of primers used in the study. (PDF)

S2 Table Segregation analysis of the transgenic tobacco expressing *EcBHLH57* transgenic plants. (PDF)

S3 Table Table showing the amplicon length, PCR efficiency and R^2^ value for the genes analyzed by qRT-PCR. (PDF)

S4 Table Comparison of yield parameters of the *EcbHLH57* expressing transgenic and wild type plants under long-term salinity stress. (PDF)
