# Supplementary material for: Overexpression of EcbHLH57 Transcription Factor from Eleusine coracana L. in Tobacco Confers Tolerance to Salt, Oxidative and Drought Stress
Source: PLoS One. 2015 Sep 14;10(9):e0137098. doi: 10.1371/journal.pone.0137098 (PMC4569372; doi:10.1371/journal.pone.0137098)
Supplement: S1 Fig — The length of nucleotide sequence is 1050 bp, sequence in brown- 5’UTR (1–99 bp); black- coding region (771 bp, 100–870 bp); green- 3’UTR (871–1050 bp); blue- polyA signal (PDF) [file pone.0137098.s001.pdf]

GAATCCCCAATTCCCGACGCGGCAGCGGAACCCTAGCACCACCCTCCTTCTACCACCTG
CGCGCGGGCTCCGGCGAGCTCCCCTCCCGCCGCCGCCACCATGACCTCCTCGGAGGGCT
CCCAGTGGGTCTTCGATTGCCCCCTGATAGATGACCTCTCGGCCGCCGACTTCGCCGCC
ACACCCGCGGGGGCTTCTACTGGACCCCGACGCCGATGCAAGCGCAGCCGCAGCCTCA
GGTGATCCAACCGCCGTCGCAGCCCGTCGCCGCCGCGGCGCCTTCCAATCCATGCAGTG
TGGAAATCAATAGCTCTGTGGACTGTAATCAGGGAAAAGAACAGCCAGTGAACAAACGT
CCAAGGTCAGAAAGCAGTGCTCAACCAAGCACAAAAGCATGTCGAGAAAAAATTAGAAG
GGACAAGCTAAATGAGAGATTCTTGGAATTGGGTGCCATTTTGGAGCCAGGCAAGACAC
CTAAAATGGACAAGTCAGCTATACTGAATGATGCGATTCGTGTAGTATCTGAATTGCGT
ACTGAAGCACAAAAGTTGAAAGATTCAAATGAGAGTTTACAAGAGAAGATCAAAGAGTT
GAAGGCTGAGAAGAATGAGCTGAGGGAGGAGAAGCAAAGGCTGAAGGCAGAAAAGGAGA
ACTTGGAGCAGCAAATAAAGTTCATGAATGCACGCCCAAGCCTTGTACCACATCCTCCT
GTTATCCCAGCATCTGCATTCACCGCACCTCAAGGACCCGCAGCAGGGCAGAAGCTAAT
GATGCCTGTGATCGGCTACCCTGGATTTCCGATGTGGCAATTCATGCCGCCTTCAGATG
TGGATACCTCTGATGACCCCAAGTCATGCCCTCCTGTTGCATAAGCAAGCGCAAGCCAG
TTGCCTGATCTTTTGGAAAGAGGCGATACTGCCCTCCATTCAAGTCTAGTCTGGTTCTC
AGTGTTGTTGATGTAGTTGACTCGAGAACTTAAGGGAAGCTAAATGTATCAGCTGGTTA
CATGGGTGATCTGACGACACCGTTGTATATTATTAGTAAATAAAATC

S1 Figure: Full-length nucleotide sequence of *EcbHLH57*. The length of nucleotide sequence is 1050bp, sequence in brown- 5’UTR (1-99bp); black- coding region(771bp, 100-870bp); green- 3’UTR (871-1050bp); blue- polyA signal.
