# Supplementary material for: Overexpression of EcbHLH57 Transcription Factor from Eleusine coracana L. in Tobacco Confers Tolerance to Salt, Oxidative and Drought Stress
Source: PLoS One. 2015 Sep 14;10(9):e0137098. doi: 10.1371/journal.pone.0137098 (PMC4569372; doi:10.1371/journal.pone.0137098)
Supplement: S2 Fig — A) Phylogenetic analysis of EcbHLH57 with closely related members of bHLH family. Multiple sequence alignment was performed using ClustalW and phylogenetic tree was constructed. B) Alignment of deduced amino acid sequences of bHLH genes. box- bHLH domain, *- leucine residues (PDF) [file pone.0137098.s002.pdf]

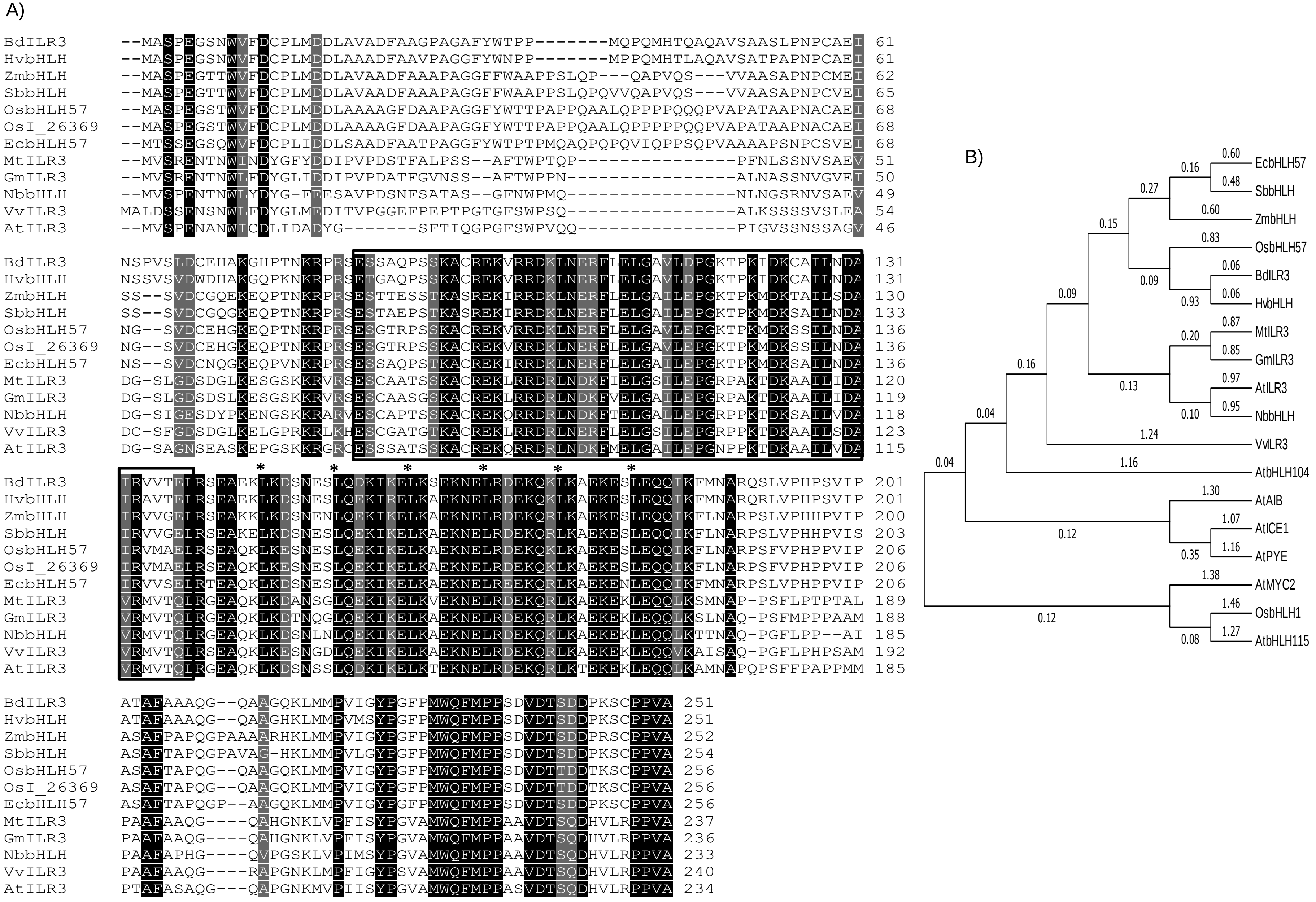

S2 Figure: Comparison of EcbHLH57 with its close homologs. A) Alignment of deduced amino acid sequences of bHLH genes. box- bHLH domain, *- leucine residues. B) Phylogenetic analysis of EcbHLH57 with closely related members of bHLH family. Multiple sequence alignment was performed using ClustalW and phylogenetic tree was constructed.
