# Supplementary material for: Overexpression of EcbHLH57 Transcription Factor from Eleusine coracana L. in Tobacco Confers Tolerance to Salt, Oxidative and Drought Stress
Source: PLoS One. 2015 Sep 14;10(9):e0137098. doi: 10.1371/journal.pone.0137098 (PMC4569372; doi:10.1371/journal.pone.0137098)
Supplement: S3 Fig — A) Physical interaction. B) Co-expression (PDF) [file pone.0137098.s003.pdf]

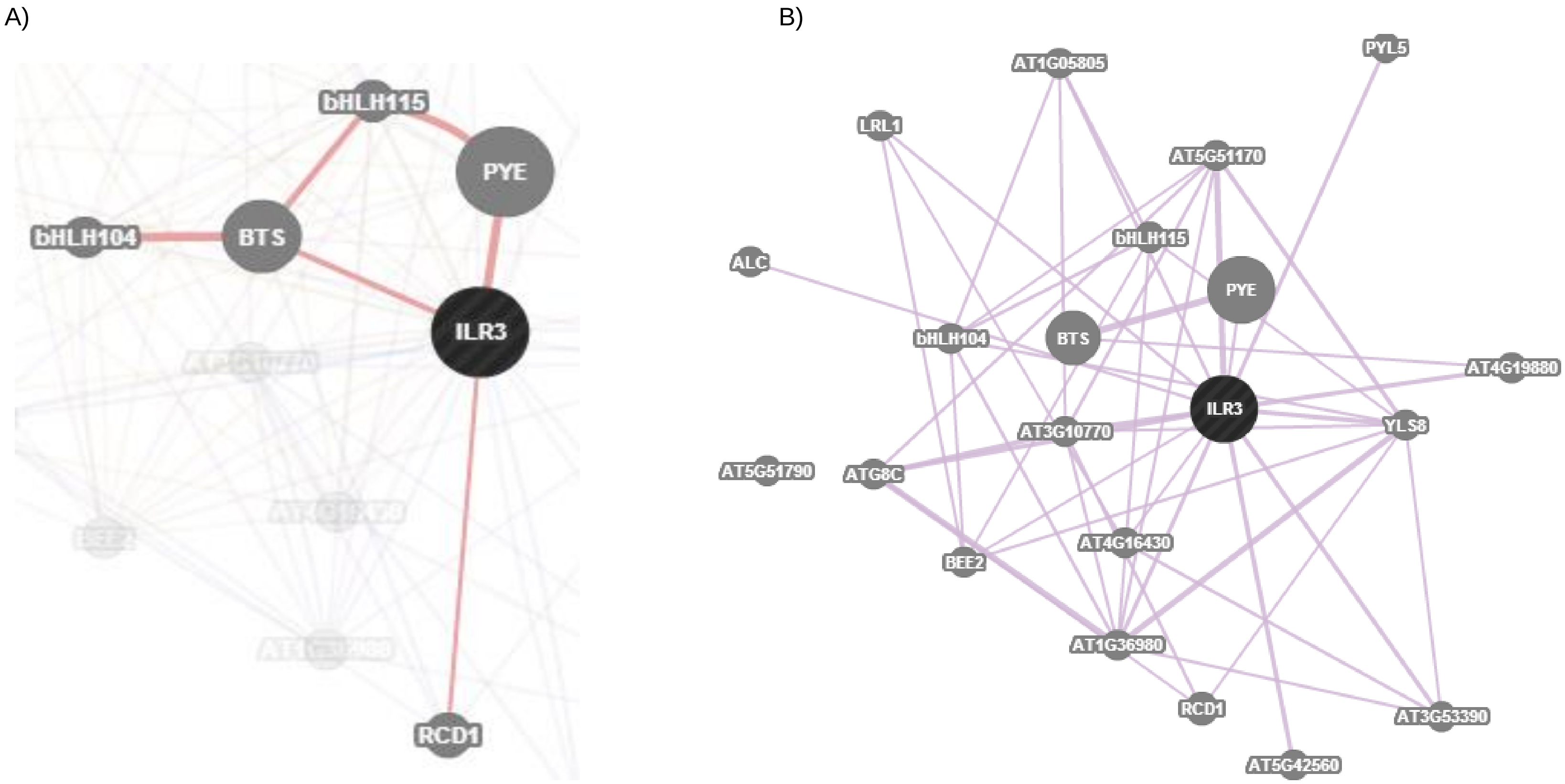


**S3 Figure: Interaction network for EcbHLH57 ortholog in *Arabidopsis thaliana* (AtILR3).** A) Physical interaction. B) Co-expression

Ilr3- transcription factor ILR3, PYE -transcription factor bHLH47, BTS- zinc finger protein-like protein, RCD1- WWE protein-protein interaction domain family protein, bHLH115- transcription factor bHLH115, AT1G05805- transcription factor bHLH128, AT1G36980- uncharacterized protein, LRL1-transcription factor bHLH66, AT5G51170- uncharacterized protein, AT3G10770- Single-stranded nucleic acid binding R3H protein, ALC- transcription factor ALC, ATG8C- autophagy-related protein 8c, bHLH104- transcription factor bHLH104, AT4G16430- transcription factor bHLH3, YLS8- mRNA splicing factor, thioredoxin-like U5 snRNP, BEE2-transcription factor BEE 2, AT5G51790- transcription factor bHLH120, AT5G42560- HVA22-like protein, AT4G19880- Intracellular chloride channel-like protein, PYL5- abscisic acid receptor PYL5, AT3G53390- transducin/WD40 domain-containing protein.
