# Supplementary material for: Overexpression of EcbHLH57 Transcription Factor from Eleusine coracana L. in Tobacco Confers Tolerance to Salt, Oxidative and Drought Stress
Source: PLoS One. 2015 Sep 14;10(9):e0137098. doi: 10.1371/journal.pone.0137098 (PMC4569372; doi:10.1371/journal.pone.0137098)
Supplement: S4 Fig — Graph depicting qRT-PCR analysis of cell-cycle regulating genes. (PDF) [file pone.0137098.s004.pdf]

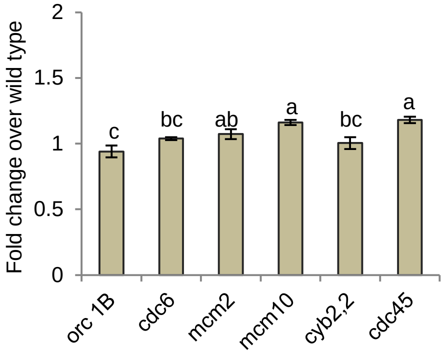

S4 Figure: Expression analysis of cell-cycle regulating genes in *EcbHLH57* expressing transgenic plants under normal growth conditions. Graph depicting qRT-PCR analysis of cell-cycle regulating genes. Data represent mean of three replications (n = 3) and bars indicate standard error. The lowercase letters that are different indicate significant difference (Duncan's multiple range test, P<0.05) between means of analyzed genes.
