# Supplementary material for: Overexpression of EcbHLH57 Transcription Factor from Eleusine coracana L. in Tobacco Confers Tolerance to Salt, Oxidative and Drought Stress
Source: PLoS One. 2015 Sep 14;10(9):e0137098. doi: 10.1371/journal.pone.0137098 (PMC4569372; doi:10.1371/journal.pone.0137098)
Supplement: S5 Fig — Germinated seeds of transgenic and wild type plants were placed on half MS media supplemented with 100 mM NaCl. i) Growth comparison of transgenic and wild type seedlings on 100 mM NaCl induced stress. ii) Fresh weight of seedlings after stress period. (PDF) [file pone.0137098.s005.pdf]

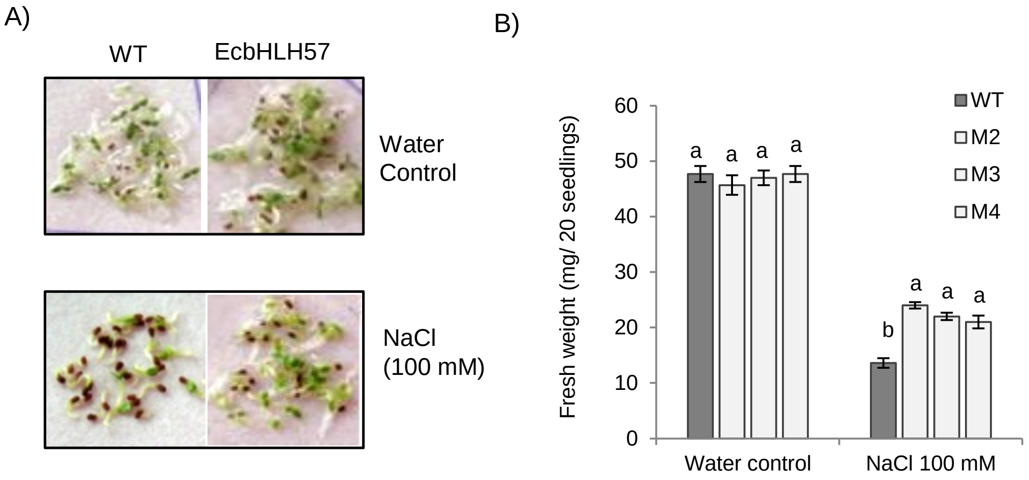

S5 Figure: Salinity stress response of *EcbHLH57* expressing transgenic plants. Germinated seeds of transgenic and wild type plants were placed on half MS media supplemented with 100 mM NaCl. A) Growth comparison of transgenic and wild type seedlings on 100 mM NaCl induced stress. B) Fresh weight of seedlings after stress period. Data represent mean of three replications (n = 3) and bars indicate standard error. The lowercase letters that are different indicate significant difference (Duncan's multiple range test, $P<0.05$) between transgenic and wild type plants exposed to same treatment.
