# Supplementary material for: Overexpression of EcbHLH57 Transcription Factor from Eleusine coracana L. in Tobacco Confers Tolerance to Salt, Oxidative and Drought Stress
Source: PLoS One. 2015 Sep 14;10(9):e0137098. doi: 10.1371/journal.pone.0137098 (PMC4569372; doi:10.1371/journal.pone.0137098)
Supplement: S6 Fig — 30-day-old plants were subjected to drought stress by withholding water for 1 week. A) Phenotype of plants under drought stress. B) Percent reduction in chlorophyll content C) Relative water content (%) D) Electrolyte leakage and E) MDA content in drought stressed plants. (PDF) [file pone.0137098.s006.pdf]

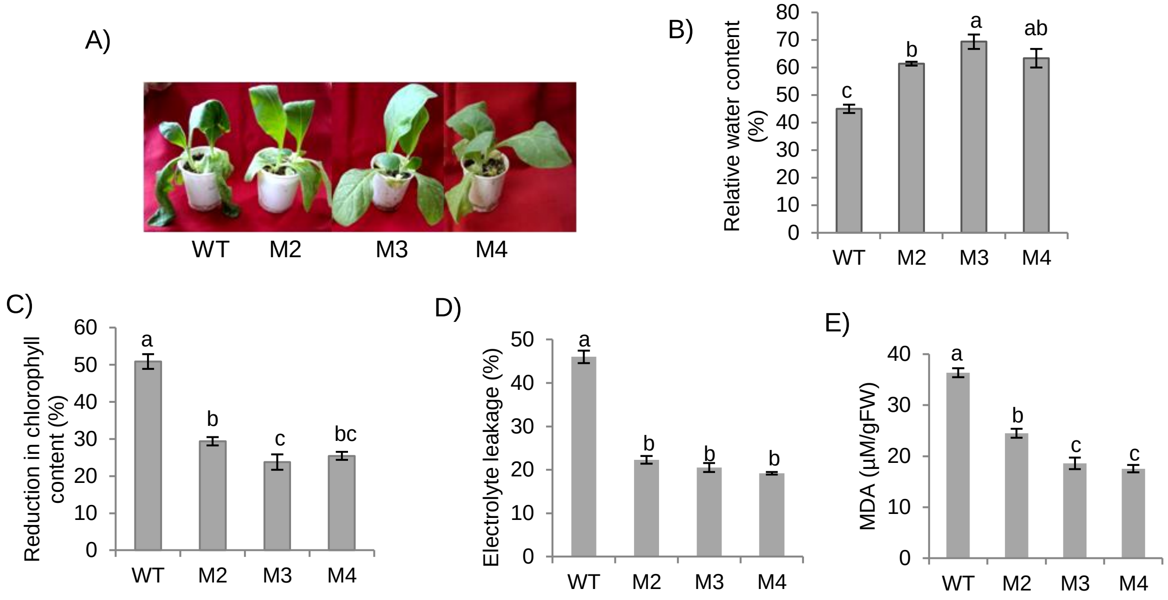

S6 Figure: Response of *EcbHLH57* expressing tobacco transgenic to drought stress. 30-day-old plants were subjected to drought stress by withholding water for 1 week. A) Phenotype of plants under drought stress. B) Percent reduction in chlorophyll content C) Relative water content (%) D) Electrolyte leakage and E) MDA content in drought stressed plants. Data represent mean of three replications (n = 3) and bars indicate standard error. The lowercase letters that are different indicate significant difference (Duncan's multiple range test, $P<0.05$) between transgenic and wild type plants exposed to same treatment.
