# Supplementary material for: Overexpression of EcbHLH57 Transcription Factor from Eleusine coracana L. in Tobacco Confers Tolerance to Salt, Oxidative and Drought Stress
Source: PLoS One. 2015 Sep 14;10(9):e0137098. doi: 10.1371/journal.pone.0137098 (PMC4569372; doi:10.1371/journal.pone.0137098)
Supplement: S1 Table — (PDF) [file pone.0137098.s007.pdf]

S1 Table: List of primers used in the study

Sl.No.	Primer name	Sequence (5’-3’)
1.	EcbHLHFP1	*gaatccccaattcccgacgcggcag*
2.	EcbHLHRP1	*gcatgctttgtgcttggttgagcaccgc*
3.	EcbHLHFP2	*gcatgccgagagaagattagaag*
4.	EcbHLHRP2	*gattttatttactaataatatacaacggtg*
5.	EcbHLHCDS F	*atgacctcctcggagggctcccagtgg*
6.	EcbHLHCDS R	*cctctttccaaaagatcaggcaactggc*
7.	EcbHLHRT F	*agagttgaaggctgaaaagaatgag*
8.	EcbHLHRT R	*ccacatcggaaatccagggtag*
11.	NTRD29AF	*tcggtgtaccaacaggcata*
12.	NTRD29AR	*cccttgctttggtgttgttt*
13.	NtLTP4F	*atgctgcagtgggattaagg*
14.	NtLTP4R	*agcagtcaatggaagggcta*
15.	NtLEA14F	*ctccgttcccgtacctatca*
16.	NtLEA14R	*caatctgcgccaatatcctt*
17.	NtP5CS F	*tggcactctctttcatcgtg*
18.	NtP5CS R	*agcttcattttcagccagga*
19.	Ntrd29B F	*tcggtgtaccaacaggcata*
20.	Ntrd29B R	*cccttgctttggtgttgttt*
21.	NtPP2CF	*agccgatgcatacagccatacaga*
22.	NtPP2CR	*caaacgcacgggaaacagcaagta*
23.	NtERD1F	*gccatgcatgaagtgatcttggca*
24.	NtERD1R	*acaaatggctgcaacagcctcatc*
