# Supplementary material for: Overexpression of EcbHLH57 Transcription Factor from Eleusine coracana L. in Tobacco Confers Tolerance to Salt, Oxidative and Drought Stress
Source: PLoS One. 2015 Sep 14;10(9):e0137098. doi: 10.1371/journal.pone.0137098 (PMC4569372; doi:10.1371/journal.pone.0137098)
Supplement: S2 Table — (PDF) [file pone.0137098.s008.pdf]

S2 Table: Segregation analysis of the transgenic tobacco expressing *EcBHLH57* transgenic plants.

Lines	Total number of seeds	Kanamycin positive/ green seedlings	Kanamycin negative /yellowing seedlings	Segregation ratio Kan^r/Kan^s	Chi-square analysis
M2	69	51	18	2.97:1	0.011
M3	93	73	20	3.13:1	0.563
M4	97	78	19	3.22:1	1.681
