# Supplementary material for: Overexpression of EcbHLH57 Transcription Factor from Eleusine coracana L. in Tobacco Confers Tolerance to Salt, Oxidative and Drought Stress
Source: PLoS One. 2015 Sep 14;10(9):e0137098. doi: 10.1371/journal.pone.0137098 (PMC4569372; doi:10.1371/journal.pone.0137098)
Supplement: S3 Table — (PDF) [file pone.0137098.s009.pdf]

S3 Table: Table showing the amplicon length, PCR efficiency and R^2 value for the genes analyzed by qRT-PCR

Sl.No.	Gene name	Amplicon length (bp)	Reaction efficiency	R^2 value
1.	*EcbHLH57*	220	1.973	1.00
2.	*FmActin*	214	1.994	1.00
3.	*NTRD29A*	203	1.928	1.00
4.	*NtLTP4*	185	1.896	0.99
5.	*NtLEA14*	183	1.945	1.00
6.	*NtP5CS*	196	1.968	1.00
7.	*Ntrd29B*	201	1.974	1.00
8.	*NtPP2C*	193	1.965	1.00
9.	*NtERD1*	204	1.922	0.99
10.	*NtSOD*	209	1.986	1.00
11.	*NtAPX*	233	1.949	0.99
12.	*NtADH1*	218	1.916	1.00
13.	*NtDREB2A*	208	1.957	1.00
14.	*NtNAC102*	197	1.991	1.00
15.	*NtELF*	188	1.964	1.00
16.	*NtORC1B*	202	1.942	1.00
17.	*NtCDC6*	186	1.899	1.00
18.	*NtMCM2*	193	1.992	1.00
19.	*NtMCM10*	216	1.938	0.99
20.	*NtCYB2,2*	207	1.957	0.99
21.	*NtCDC45*	189	1.982	1.00
