# Supplementary material for: Overexpression of EcbHLH57 Transcription Factor from Eleusine coracana L. in Tobacco Confers Tolerance to Salt, Oxidative and Drought Stress
Source: PLoS One. 2015 Sep 14;10(9):e0137098. doi: 10.1371/journal.pone.0137098 (PMC4569372; doi:10.1371/journal.pone.0137098)
Supplement: S4 Table — (PDF) [file pone.0137098.s010.pdf]

S4 Table: Comparison of yield parameters of the *EcbHLH57* expressing transgenic and wild type plants under long-term salinity stress. Data represent mean of three replications (n = 3) and bars indicate standard error. The lowercase letters that are different indicate significant difference (Duncan's multiple range test, P<0.05) between transgenic and wild type plants exposed to same treatment.

Lines	Plant height (cm)	TDM (g)	Seed weight/pod (mg)	Number of pods/plant (number)
WT	64.5 ± 2.1^{c}	29.9 ± 0.6^{c}	87.0 ± 4.0^{c}	16.0 ± 1.0^{c}
M2	85.9 ± 1.6^{b}	44.0 ± 1.3^{b}	117.0 ± 3.0^{b}	34.5 ± 2.5^{b}
M3	100.3 ± 2.6^{a}	52.0 ± 1.1^{a}	126.5 ± 2.5^{a}	41.0 ± 3.0^{a}
M4	91.0 ± 2.8^{ab}	50.5 ± 1.0^{a}	123.0 ± 2.0^{a}	38.5 ± 3.5^{ab}
